# Stakeholders' perspectives on the regulation and integration of complementary and alternative medicine products in Lebanon: a qualitative study

**DOI:** 10.1186/1472-6882-11-71

**Published:** 2011-08-28

**Authors:** Mohamad Alameddine, Farah Naja, Sarah Abdel-Salam, Salwa Maalouf, Claudia Matta

**Affiliations:** 1 Faculty of Health Sciences, Department of Health Management and Policy, American University of Beirut. Lebanon; 2 Faculty of Agriculture and Food Sciences, Department of Nutrition and Food Sciences, American University of Beirut. Riad El-Solh, Beirut, 1107 2020, Lebanon. +961-350000- extension 4504. Lebanon; 3 Department of Family Medicine, American University of Beirut Medical Center. Lebanon

## Abstract

**Background:**

The regulation of the markets for Complementary and Alternative Medicine (CAM) products presents a global challenge. There is a dearth of studies that have examined or evaluated the regulatory policies of CAM products in the Eastern Mediterranean Region (EMR). We investigate the regulatory frameworks and the barriers for the proper regulation and integration of CAM products in Lebanon, as an example of an EMR country with a weak public infrastructure.

**Methods:**

We utilized a qualitative study design involving a series of semi-structured interviews with stakeholders of the CAM market in Lebanon. Snowball sampling was used to identify interviewees; interviews continued until the "saturation" point was reached. A total of 16 interviews were carried out with decision makers, representatives of professional associations, academic researchers, CAM product importers, policy makers and a media representative. Interviews were transcribed and thematic analysis of scripts was carried out.

**Results:**

There was a consensus among all stakeholders that the regulation of the market for CAM products in Lebanon needs to be strengthened. Thematic analysis identified a number of impediments jeopardizing the safety of public consumption and hindering the integration of CAM therapies into mainstream medicine; including: weak infrastructure, poor regulation, ineffective policies and politics, weak CAM awareness and sub-optimal coordination and cooperation among stakeholders. With respect to policy instruments, voluntary instruments (self regulation) were deemed ineffective by stakeholders due to poor awareness of both users and providers on safe use of CAM products. Stakeholders' rather recommended the adoption of a combination of mixed (enhancing public awareness and integration of CAM into medical and nursing curricula) and compulsory (stricter governmental regulation) policy instruments for the regulation of the market for CAM products.

**Conclusions:**

The current status quo with respect to the regulation of CAM products in Lebanon is not conducive to public safety, nor does it support the integration of CAM products into the healthcare system. The Ministry of Health indeed plays a dominant role in the regulation of these products through a combination of mixed and compulsory policy instruments. Yet, the proper implementation of these regulations requires political resolve coupled with the cooperation of all CAM stakeholders.

## Background

Complementary and Alternative Medicine (CAM) are a diverse group of medical and healthcare systems, practices and products that are not considered part of conventional medicine, yet complement it by diversifying the conceptual frameworks of medicine or by satisfying a demand not met by orthodoxy [1,2]. The US National Center for CAM therapies divides CAM into four categories: (1) Mind-body systems; (2) Manipulative and body-based practices; (3) Energy Medicine; and (4) ***Biologically based practices ***[1]. In this manuscript, CAM refers to biologically based practices including substances found in nature, such as herbs, dietary supplements, multivitamin and mineral supplements [1,3]. The increasing popularity and usage of CAM products has precipitated regulatory challenges for many countries across the globe [4-11]. In Lebanon, the number of CAM products introduced into the market grew steadily since 1980. In 2003, the total number of CAM products in the Lebanese market was estimated at around 1,300 [12]. Recent estimates indicate that the number of CAM products has exceeded 3000. Amid the increased popularity of CAM products and the growing size of their market [13,14], there is a dearth of systematic studies investigating the regulatory framework for CAM products and the barriers towards their proper integration into the healthcare system in the Eastern Mediterranean Region (EMR).

### The argument for regulation

Regulation of CAM products is geared towards ensuring quality standards and enhancing consumers' safety [15]. Without proper regulation, CAM users could be subjected to various degrees of risks ranging from inappropriate use to serious life threatening adverse events [16]. Various jurisdictions have reported an issue with the labeling and advertising of CAM products; false claims of a 'magic treatment' are not uncommon [17]. Governmental interventions are necessary to protect customers against false and misleading claims; possible manipulation of safety test results; substandard manufacturing processes and substitution of ingredients [18].

Although the majority of CAM products may not carry any side effects to consumers, advertised false claims could cause consumers to modify or stop the use of essential prescribed medications without proper medical advice. This jeopardizes the safety of consumers and places a serious mandate on governments to lead a regulatory agenda. Furthermore, acute or chronic overdose has been reported in individuals falsely believing that increased consumption of the 'All Natural' CAM products will enhance their acclaimed benefits and lead to faster recovery or relief [19]. Proper regulation is also necessary to protect against biological interactions between CAM products or between CAM products and pharmaceuticals [19].

Note that the cost of CAM products in the EMR is usually covered out of pocket, since private or social insurance programs do not cover such costs. Therefore, improper utilization of CAM products could cause a financial burden on the stretched resources of poor and middle income families. Furthermore, using CAM products could delay the use of orthodox medicine which increases the burden of disease on governments or individuals, who might incur greater costs to cure once easy-to-manage diseases [19]. In conclusion, in the absence of regulation the use of CAM products may harm consumer either directly through adverse events, or indirectly by creating an unwarranted financial and emotional burden on users [20].

Governmental regulation is pivotal to protect against these harms and could facilitate the integration of CAM products into modern healthcare systems. While regulation of the CAM market presents a challenge for many countries across the globe, regulation is particularly challenging in countries with a weak public sector; such as Lebanon [21]. Regulatory policy instruments, at the discretion of governments, can be arranged along a scale depending on the degree of 'legitimate coercion' involved [22]. According to Doern and Phidd (1992), at one end of this scale are voluntary instrument, the least coercive of which is 'self regulation'; while at the other end lies compulsory instruments, the most coercive of which is the complete 'public ownership' of markets or resources. In between the two extremes lie a number of mixed policy instruments involving various degrees of public and private regulation, including: public education, subsidization and product taxation [22].

### CAM regulation and integration: a global challenge

A plethora of factors make CAM regulation and integration a global challenge. These factors include absence of guidelines for CAM practice, insufficient research to prove cost-effectiveness and the limited commitment of insurers for reimbursement [23,24]. Other challenges related to CAM providers include: ignorance about CAM, lack of licensing for CAM professionals, fear and resistance to change by medical establishment, lack of data on usage of CAM, lack of provider networks, as well as lack of coordination and communication between CAM providers and the existing health systems [24,25].

In its global survey report on national policies on traditional medicine and regulation of herbal medicines, WHO stated that the major challenges are related to "regulatory status, assessment of safety and efficacy, quality control, safety monitoring and lack of knowledge about TM/CAM" [26].

### CAM products regulation in the Eastern Mediterranean Region

Although the Eastern Mediterranean Region hosts one of the fastest growing markets of CAM products in the world [27], little is known about the use of CAM products in the region, except for a number of sporadic reports that have examined specific patient populations [28,29] or shy attempts to examine prevalence of use in the Arabian Gulf region [30,31]. In addition, little is published with respect to the regulation of CAM products in these countries.

### CAM regulation in Lebanon

In lights of the growing market for CAM products in Lebanon, the Lebanese Ministry of Health (MOH) assigned an expert committee that was responsible for regulating the entry of CAM products into the Lebanese market. The committee received approval from the cabinet of Ministers in 1998 (decree number 11710). The committee, led by the Director General of the MOH, includes seven members from the trade, education and research communities. The recommendations of this committee receive final approval from the Minister of Health.

**Figure 1:**
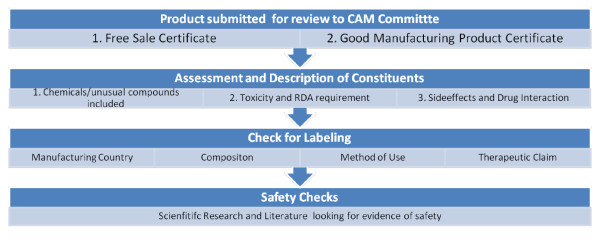
**The CAM Review Process in Lebanon**.

The mandate of the expert committee included the assessment of requests presented to the MOH for the import, distribution and marketing of CAM products. For example imported CAM products have to be certified for sales by the concerned ministries in their country of origin (free sales certificate) and should present a Good Manufacturing Practice (GMP)-certificate summarizing the content of the manufacturing license. The companies should further present a detailed description of constituents and a copy of the package label for each of the products to be reviewed & approved prior to entry into the Lebanese market. In the absence of this review and approval process, products are prohibited from the entry into the Lebanese market and their sale is considered illegal. An illustration of the CAM review process in Lebanon is presented in Figure 1.

In contrast to CAM, OTC products in Lebanon are dealt with as pharmaceutical products and are thus subjected to a much more stringent review and approval processes. In addition to the requirements stated for CAM products, the certification and review process for OTC products requires importers to present a certificate of pharmaceutical product, data on purchasing price and public price, certificate of analysis and stability data. OTC importers should also present a complete technical file, including information on the product's pharmacology, physiochemical properties, bioavailability, teratogenicity, inhibility tests for antibiotics, sterility and progenicity, clinical studies and an extensive supportive biography.

Despite the fast and steady growth of the number of CAM products and outlets in Lebanon and amid a growing public rhetoric on absent or ineffective regulation of this market, our literature search reveals a dearth of studies that have examined the regulatory policies of CAM products and their implementation in the EMR countries, in general, and Lebanon in particular.

### Objectives

This study aims at soliciting the feedback of stakeholders on the barriers for the proper regulation and integration of CAM products in the Lebanese healthcare system.

## Methods

### Ethical approval

The proposal, interview schedule and consent form for this study was reviewed and approved by the Institutional Review Board at the American University of Beirut.

### Study design

We utilized a qualitative study design carrying out a series of key informant interviews with stakeholders of the CAM market in Lebanon. Well-conducted key informant interviews can provide a desirable combination of objectivity and depth and often generate valuable data that could not be successfully obtained by another approach. Interviews can further allow for diverse perspectives on the events and fill in any documentation gaps [32].

### Selection of interviewees

**Figure 2:**
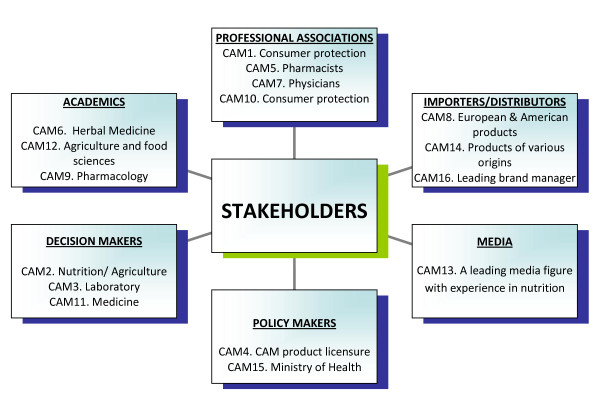
**Distribution of Stakeholders by type and background**.

In the choice of interviewees, we aimed at investigating the different roles and perspectives of the multiple stakeholders in the CAM field. Interviewees in this study included decision makers, representatives of professional associations (Heads of orders and syndicates), academic researchers (senior academicians from faculties of agriculture, nutrition and pharmacy), CAM product importers (owners or companies and CAM product importers), policy makers (senior managers at the Ministry of Health and Ministry of Trade) and a media representative (a public figure familiar with the field). A more detailed matrix of the interviewees, showing the background of each of the stakeholders, is presented in Figure 2.

### Interview schedule

A semi-structured interview schedule of ten questions was utilized for data collection in this study. The interview schedule was developed by the research team to investigate the types of CAM products available in the market in Lebanon, the regulatory frameworks in place and the ways through which they can be enhanced, and the barriers to the integration of CAM products into the Lebanese Health Care system. Interviewees were further asked to share with the research team any relevant documents and to suggest the names of other stakeholders that could contribute to this study.

### Data collection

A number of key stakeholders (an academic, policy maker, importer and a representative of a professional association) were initially identified and invited to participate in the study. Snowball sampling was subsequently used to identify additional stakeholders. This process of interviewing was continued until the "saturation" point was reached; i.e., until no new information altered the results already obtained and the names identified by interviewees for additional interviews were repeated [33].

All key informants were sent a standard email explaining the objectives of the study and inviting them to a personal interview. The consent form and the interview schedule, approved by the American University of Beirut Institutional Review Board (ethics), were attached to the invitation. When potential interviewees did not respond to the email within one week, the research team followed up with a phone call. Overall, 16 out of a total 19 key informants accepted the invitation to an interview and signed the consent form (84.2%). The three key informants that did not accept to be interviewed apologized due to their busy work/travel schedule.

All interviews were conducted in the spring of 2010. Interviews lasted an average of one hour. At the beginning of each interview, interviewees were asked to sign the consent form, which also included details about their rights as participants in this research and acquired their approval for tape recording the interviews. All interviews, but one, were tape recorded. Along with tape recording, the interviewers took field notes to facilitate data analysis and provide a backup for important findings in case certain data elements were missed during the taping of interviews.

### Data analysis

Thematic analysis was utilized in analyzing the data collected from the key informant interviews. In thematic analysis, researchers seeks themes that emerge from the interview narratives through an ongoing process that requires them to detect recurring issues and patterns from the data rather than from predetermined codes [34-36].

The interview tapes were then transcribed by the research team. The transcripts provided an accurate text of the interviews with "XXX" placed whenever the discussion was not clear and capital letters to emphasize intensity of certain points. Each transcript was first read thoroughly by two members of the research team. Initial readings of the scripts were meant to gain a 'primary feeling' of the text and a general insight into the answers of the interviewees. As a first step, each of the questions of the interview script was used as a broad theme and the answers of the interviewees were classified under it. The statements that did not fit under any of these questions were allocated to a theme titled 'miscellaneous'.

After the primary coding of all transcripts, analysis sheets were re-coded into comprehensive broad themes and subthemes by the two members of the research team. Any coding disagreements between the two were resolved in a series of research meetings until a final list of sub-themes were generated. The final analysis sheet included the newly formulated broad themes and their corresponding subthemes. Relevant quotes were collated under the various themes/sub-themes. All the members of the research team reviewed and approved of the final list of themes and sub-themes.

## Results

Data analysis of stakeholders' interviews identified three overarching themes (and twelve associated sub-themes) describing the market for CAM products in Lebanon, current regulatory mechanisms and obstacles to proper regulation/integration. These three themes were: Market characterization, critique of current regulation and barriers to proper regulation/integration. They are discussed below, along with their respective sub-themes, in further details.

### Market characterization

Key informants' characterization of the market for CAM products in Lebanon was listed under the theme of "Market Characterization". This theme included a general description of the market and associated forces shaping products' supply and demand. Sub-themes included: CAM products' database, supply challenges, common categories and triggers of consumptions of CAM products.

There was a consensus among interviewees that it is difficult to produce a comprehensive database of all the products available in the Lebanese market. This could be attributed to several supply challenges including: the large number of CAM products that already exist at Lebanese market and the loop holes in the system that allow the entry of CAM products through a diversity of official and unofficial channels that are beyond the control of the Ministry of Health (MOH).

With respect to CAM products' categorization, interviews revealed that CAM products in the Lebanese market could be collated into four major categories: Weight loss products, energy/sexual enhancers, body/muscle building and multivitamins.

Furthermore, interviewees outlined several triggers of consumption of CAM products, the most important of which were: loss of hope for a cure using orthodox medicine; frustration with side-effects of orthodox medicine; the relatively simpler use and lower cost of CAM products (no medical prescription needed, no doctor's fee and CAM products could be delivered to one's door); perceived benefits of CAM products since they are all natural products; and promises of a fast cure propagated by media sources.

### Critique of current regulation

The "critique of current regulation" theme summarizes the CAM stakeholders' perspectives on the regulation of CAM products and includes three main sub-themes: the entry venues of CAM products into the market, the review process, and the selling processes of CAM products in the Lebanese market.

Most stakeholders agreed that the CAM sector in Lebanon is poorly regulated. This is not only due to the lack of policies, procedures and regulations, but also due to the chronic lack of adherence to existing regulations. Originally, importers of CAM products had to pledge not advertise their products on television, to restrict their sales in pharmacies and not to make any therapeutic claims for these products. Yet, over time, and due to multiple factors including the poor monitoring of the CAM market and the poor compliance to regulation, CAM products became difficult to regulate. This was eloquently stated by one of the interviewed key informants:

"The law as it stands today is not the biggest problem; it is rather the compliance to existing regulations and the need to update and strengthen these regulations"

### The product entry process

Interviewed stakeholders voiced a concern with the poorly enforced entry process of CAM products into the Lebanese market. The official steps for the entry of CAM products to the Lebanese market are well documented, including: entry & review of samples, submission to review committee at the MOH, review of the product by the expert committee at the MOH, and finally registration or rejection of the product. Yet, according to stakeholders, poor adherence to the documented MOH policy coupled with loop holes in the regulations that restricts the entry of CAM products through other venues, significantly eroded the effectiveness of current regulation. Stakeholders stated the following entry venues for CAM products: through the Ministry of Economics and Trade as food supplements; with a special permission from the Minister of Health, or by smuggling the product into the Lebanese territories. The quotes below demonstrate this point:

"What bothered us a lot is that we would decide on forbidding certain products but the Minister would still allow them."

"Things are entering from beneath the table and above the table. Who is responsible? No one is!"

### The review process

Interviewees were divided on whether the review requirements are enough and whether laboratory analyses might be necessary. For many, proper regulation of CAM products is necessarily correlated with laboratory testing because it would be the only way to assure the content of the products entering the market. Concerns were expressed over changing the labels of the products that were refused on a first review and submitting them again for a second review; using the words of one of the interviewed stakeholders:

"The system was imperfect indeed. Sometimes we discovered that certificates/labels presented to us were fake and that companies did not exist."

A number of stakeholders also pinpointed that the CAM review process could be strengthened by revising the membership of the CAM review committee to include members that have better expertise in the field of CAM.

### The selling process

A consensus was reached among stakeholders that the selling practices are rather erratic and in urgent need of regulation and control. The current status quo is not conducive to consumer protection. For example, as expressed by one of the interviewees:

"Some products might be good products but advertised for in the wrong way and sold for the wrong reasons."

Furthermore, interviewees agreed that there is no control on the market pricing of CAM products and that the price of some products is unjustifiably high. It was also established that each importer sets the price they desired for their products with no governmental control and that some products are seriously overpriced because of the costs of advertising. One might think that, according to basic economics, the higher price of the product would decrease its demand, yet this is not the case in Lebanon. This is primarily because consumers are led by intense advertising to think that higher priced products are more beneficial. The quote below highlights this issue:

"Although their prices are high, when people see something on TV and when you tell them it is expensive, then they think it is better"

There was also disagreement as to which authority should ideally regulate the pricing of CAM products. A number of stakeholders believe that product pricing should be the responsibility of the MOH while others designated the Ministry of Economics as the decision maker in terms of pricing.

### Barriers to proper regulation/integration

Thematic analysis of stakeholders' responses revealed a number of concerns related to the barriers which are impeding the proper regulation and integration of CAM products in the Lebanese market. These barriers are categorized under five main sub-themes: weak infrastructure, poor regulation, negative role played by politics, weak public/provider awareness and sub-optimal coordination and cooperation among stakeholders.

With respect to infrastructure, one of the major limitations in scaling up the control, regulation and integration of CAM products is the absence of a central laboratory and hence, the absence of resources to test the products that are entering the market. In addition, a database for approved CAM products is nonexistent due to the shortage of human resources at the MOH and the general lack of required expertise in Lebanon. This makes the analysis and follow-up on these products beyond the capabilities of the current regulatory bodies.

Regulatory issues highlighted by stakeholders relate to the limited power delegated to the expert committee, whose decisions are advisory and not binding. This, added to the multiple entry points of CAM products, has caused gradual erosion in the leverage of decision making for the CAM expert committee. The diversity of entry means has also made it difficult to assign responsibility and accountability for the safety of the products sold in the market. Many stakeholders expressed concerns that products that were refused entry into the market by the expert committee, products that were under review or those that were never reviewed, were all being sold in the market; According to one interviewee:

"I once saw a product that was still under review on the shelf of a pharmacy. In the review, I found that it contains laxatives and should not be granted entry into the market"

Such violations have eroded the trust of various stakeholders in the credibility of the committee and have also negatively influenced the commitment of committee members.

There were recurrent calls to review the terms of reference and empower the 'CAM expert committee'. Interviewees also shared that it should be the prerogative of the committee to regulate the selling points of products and be able to implement this decision on the ground. Quoting one interviewee:

*"The MOH shoul*d *give the committee full liberty to come up with independent decisions that are BINDING*."

Interviewed stakeholders also described the negative role played by politics as a major obstacle in the regulation and integration of CAM products. Issues highlighted under this theme ranged from the general lack of political will by the government to enhance the regulation of this sector, to concerns about corruption, bribery and favoritism, and finally, to the political protection of some distributers of CAM products. Last but not least, stakeholders' described the negative role played by some media outlets; most of which were politically affiliated. For example one interviewee stated that:

"No one will stand up against the media. No one dares to do that"

Further hindering the integration of CAM into the Lebanese healthcare system was the poor awareness of both the public and the healthcare providers of CAM products. Stakeholders described an unfortunate situation were reliable information about CAM products was virtually non-existent for consumers. According to stakeholders, Lebanese CAM consumers often faced the dual disadvantage of misleading advertisements and the poor CAM literacy among healthcare providers. One of the interviewed stakeholders summarized the situation by saying:

"Unfortunately, we are in a country where there is no awareness of CAM"

Stakeholders attributed the providers' poor knowledge of CAM to the lack of integration of CAM education and training into the medical curricula and professional education programs. The quote below highlights this point:

"Physicians need education, they need to know exactly what CAM products exist (in the market), what has adverse effects. Such knowledge does not currently exist among physicians"

Last but not least, interviewed stakeholders expressed concern over the general lack of communication and coordination among the various stakeholders in the CAM market. The main identified stakeholders were the Ministry of Health, Ministry of Economics and Trade, Ministry of Interior, Ministry of Media, Consumer protection agency, the order of pharmacists, the order of physicians, academic institutions, distributers and media outlets. According to many interviewees, there was little or no collaboration between stakeholders on the regulation of the CAM market. To rectify the situation, a number of stakeholders suggested the establishment, under the patronage of the Ministry of Health, of a national CAM coordinating committee on which all stakeholders would be represented. Furthermore, the need to strengthen the role of consumer protection agencies was highlighted. The two quotes below demonstrate the view point of interviewees:

*"Allocating a bigger role to consumer protection offices would allow splitting tasks in a way that would avoid any conflict of interest"*.

"Consumer protection associations need to be more pro-active...taking the lead of a centralized office for complaints and should carry out post-market entry follow up."

## Discussion

A key decision that Lebanon and other countries in the EMR region have to make in the regulation of CAM markets is the degree of public involvement in such regulation. The current study unearthed a number of impediments that are decreasing the efficiency and effectiveness of the regulation of CAM products in Lebanon. Such impediments are consequently leading to uninformed or unsafe public consumption of these products and are hindering the integration of CAM therapies into mainstream medicine. Yet, Lebanon is not alone in facing these regulatory hurdles.

In 2005, the World Health Organization (WHO) surveyed EMR countries with respect to the national programs they have in place to regulate CAM products. Surveyed EMR countries exhibited a wide variance regarding the structure and comprehensiveness of the policies, laws and regulations on CAM products. Perhaps most telling about the poor regulatory capacity was that fact that only a third of the surveyed EMR countries had a formal coordinating body for review and regulation of CAM/Traditional Medicine products [26]. Furthermore, the fragmented healthcare systems in these countries and the lack of cooperation and information sharing between the different ministries of health contributed to the slow progress in developing national policies that facilitate the integration of CAM [37].

We believe that our overview of regulatory mechanisms in Lebanon, coupled with the stakeholders' interviews, revealed that voluntary instruments and self regulation are indeed disfavored as they are not viewed as instruments conducive to proper regulation of the CAM market in the country. This could be attributed to the poor awareness of the public on the safe use of CAM products, coupled with the lack of proper knowledge of healthcare providers on how CAM products could be integrated into the treatment protocols of their patients.

A careful synthesis of stakeholders' responses reveals an overall inclination to adopt a combination of mixed and compulsory policy instruments [22]. Specifically, stakeholders spoke about the need to enhance the awareness of both the public and the providers on the safe consumption of CAM products (a mixed policy instrument), as well as the endorsement of a stricter governmental regulation of the CAM sector (compulsory instrument). Note that while interviewed stakeholders recommended stricter governmental regulation of CAM products, none endorsed the adoption of the stringent review and approval processes that are applicable to OTC products in Lebanon (they are dealt with as pharmaceuticals).

According to stakeholders, strengthening the existing CAM regulatory mechanisms requires the reorganization of the sector to ensure a stronger governmental control through better safety and efficacy testing. Decrees, laws and legislations are indeed necessary but not sufficient for the proper regulation of the market for CAM products. Regulations need to be supplemented by effective implementation, proper awareness and control. The role of the MOH in organizing and regulating the market for CAM products could indeed be strengthened by using a systematic approach to drug regulation through the drafting and lobbying at the parliament for the passage of a national drug policy. Other suggestions that could potentially enhance CAM products' regulation include: establishing and empowering a new CAM expert committee; creating a comprehensive publically accessible database for CAM products approved for sale; ensuring (in collaboration with the Ministry of Media) the monitoring of media outlets' compliance with the guidelines for CAM advertisement; and leading awareness campaigns to promote safe consumption.

Two remarks are noteworthy regarding the above mentioned stakeholders' recommendations. The first is that most are in conformity with the ones previously published in WHO report [26,38,39]. The second relates to the applicability of many of the above recommendations to other EMR countries, who face comparable regulatory, legal and organizational challenges. For example, according to WHO, even when regulatory bodies existed in EMR countries, their functionality and effectiveness is dubious and is not necessarily conducive to integrating these products into the main stream healthcare system [26].

The need to educate the public in order to raise awareness amongst both consumers and professionals was highlighted as a mixed policy instrument that could enhance the development and regulation of the market for CAM products. It is a shared responsibility among both public and private authorities (e.g. consumer protection agency, syndicates and orders) to educate the public on the safe and effective consumption of CAM products. This could be operationalized through the organization of awareness campaigns, publishing an annual inventory of approved and safe products in a printed and electronic format, and dedicating a hotline to provide consumers with information on the safe consumption of CAM products.

Another mixed policy instrument that deems a close collaboration and coordination between the public and private sector is the integration of CAM into the medical and nursing curricula, as well as the establishment of new training programs for CAM product specialists. Such curricular review and redesign efforts should be supplemented by a professional development programs aiming at educating practicing professionals (e.g. physicians, nurses, dietitian, pharmacists, etc.) on the safe and effective use of CAM products.

A number of shortcomings in this manuscript are worth mentioning. First, although every effort has been exerted to ensure that the stakeholders are well representatives of the CAM market, a stronger voice for consumers and for the less visible community stakeholders (not known to the authors) would have been preferred, yet was not possible with the snowball sampling technique. Second, it cannot be guaranteed that stakeholders' own impressions and biases were excluded from the analysis. Yet, the authors tried to minimize such biases through identifying recurrent themes or by asking stakeholders to provide evidence for certain dubious statements. Note that extra caution was exerted when analyzing the answers of the stakeholders who have served as CAM expert committee members (three out of sixteen) in order to ensure that their answers are not self serving. Third, triangulation of the identified themes with a thorough and contextual literature review could have strengthened the validity of the findings, yet the scarcity of literature on the regulation and integration of CAM products in the EMR region did not allow for this. Finally, three of the identified stakeholders did not agree to participate due to personal or professional reasons, it could not be verified whether these stakeholders hold points of view that might be different from those that agreed to an interview.

## Conclusions

Public regulation of markets is no easy task. The diversity of policy instruments and regulatory schemes available at the discretion of policy makers, coupled with the lack of understanding of such schemes and weak public infrastructure, further impedes proper regulation in the EMR region in general, and Lebanon in particular.

Although the MOH is identified as the stakeholder playing a dominant role in CAM market regulation and licensing, proper regulation remains a shared responsibility among all stakeholders. The role of the MOH in organizing and regulating the market for CAM products could be strengthened through a combination of mixed and compulsory policy instrument, including: initiating the drafting of supporting legislations, streamlining the entry and review processes for CAM products, and spearheading public awareness initiatives.

Some of the identified impediments to proper regulation of CAM markets and identified policy instruments might be specific to the Lebanese context, yet many would certainly apply to other EMR countries. The authors acknowledge that the corrective measures and ideas suggested in this manuscript will not happen overnight nor would they have to be all implemented. Yet progress would be made if such recommendation could be integrated into a national drug policy, endorsed by all concerned stakeholders, and supported by a clear plan of action. Such a policy would aim at enhancing public safety and well-being through the proper regulation and integration of CAM products into healthcare system. Political resolve coupled with the support of all stakeholders, would be prerequisites for the success of current and future regulation and integration attempts.

## Competing interests

The authors declare that they have no competing interests.

## Authors' contributions

*MA: *Conceptualized the study, wrote the proposal, secured funding, led all aspects of data collection and analysis and prepared the first draft of the submitted manuscript. He approved the final submitted version of this manuscript; *F N: *Conceptualized the study, wrote the proposal and secured the funding. Contributed to data analysis of collected information and helped in the write up of the manuscript. She approved the final submitted version of this manuscript; *SA: *Carried out a number of the key informant interviews and helped with data transcription and thematic analysis. She reviewed the manuscript and approved the final submitted version; *SM: *Carried out a number of the key informant interviews and helped with data transcription and thematic analysis. She reviewed the manuscript and approved the final submitted version; and *CM: *Contributed to thematic analysis and helped in reviewing the manuscript. She approved the final version of the submitted manuscript.

## Pre-publication history

The pre-publication history for this paper can be accessed here:

http://www.biomedcentral.com/1472-6882/11/71/prepub

## References

[B1] NIH: National Center for Complementary and Alternative Medicine http://nccam.nih.gov/health/whatiscam/

[B2] Ernst E Cassileth BR How useful are unconventional cancer treatments? Eur J Cancer 1999 35 11 1608 1613 10.1016/S0959-8049(99)00198-7 10673970

[B3] Hoffman C Integrated Medicine Conference Report: can alternative medicine be integrated into mainstream care? Complement Ther Nurs Midwifery 2001 7 2 110 114 10.1054/ctnm.2001.0538 11855771

[B4] Morrow JD Why the United States still needs improved dietary supplement regulation and oversight Clin Pharmacol Ther 2008 83 3 391 393 10.1038/sj.clpt.6100500 18285784

[B5] Harvey KJ Korczak VS Marron LJ Newgreen DB Commercialism, choice and consumer protection: regulation of complementary medicines in Australia Med J Aust 2008 188 1 21 25 18205557 10.5694/j.1326-5377.2008.tb01905.x

[B6] Clark J Regulation of natural health products challenged CMAJ 2004 170 8 1217. 15078838 10.1503/cmaj.1040481 PMC385347

[B7] Larsen LL Berry JA The regulation of dietary supplements J Am Acad Nurse Pract 2003 15 9 410 414 10.1111/j.1745-7599.2003.tb00415.x 14560437

[B8] Choi DW Kim JH Cho SY Kim DH Chang SY Regulation and quality control of herbal drugs in Korea Toxicology 2002 181-182 581 586 12505370 10.1016/s0300-483x(02)00487-0

[B9] Walker LA Budd S UK: the current state of regulation of complementary and alternative medicine Complement Ther Med 2002 10 1 8 13 10.1054/ctim.2002.0522 12442817

[B10] Saito H Regulation of herbal medicines in Japan Pharmacol Res 2000 41 5 515 519 10.1006/phrs.1999.0645 10753549

[B11] Rousseaux CG Schachter H Regulatory issues concerning the safety, efficacy and quality of herbal remedies Birth Defects Res B Dev Reprod Toxicol 2003 68 6 505 510 10.1002/bdrb.10053 14745988

[B12] Catacora Vargas G Preliminary Market Assessment of Herbal Remedies in Lebanon Master of Science 2003 American University of Beirut, Beirut, Lebanon

[B13] Boulos Janay As use of herbal remedies rises, so does debate on their effectiveness The daily star 2008 Local news

[B14] Hodeib Mirella War of the remedies: Mainstream medicine takes on herbal upstarts The Daily Star 2007 Lebanon Examiner

[B15] Anonymous Interim progress report White House Commission on Complementary and Alternative Medicine Policy J Altern Complement Med 2001 7 6 703 713 11822619 10.1089/10755530152755261

[B16] Cohen MM Penman S Pirotta M Da Costa C The integration of complementary therapies in Australian general practice: results of a national survey J Altern Complement Med 2005 11 6 995 1004 10.1089/acm.2005.11.995 16398590

[B17] Burton B Australia backs tougher regulation for complementary health products BMJ 2005 330 7492 619. 15774981 10.1136/bmj.330.7492.619 PMC554900

[B18] Loff B McKelvie H Australia shaken by complementary medicines recall Lancet 2003 361 9370 1710. 12767742 10.1016/S0140-6736(03)13384-3

[B19] Shaw D Risks or remedies? Safety aspects of herbal remedies in the UK J R Soc Med 1998 91 6 294 296 9771511 10.1177/014107689809100602 PMC1296770

[B20] Cohen MH Kemper KJ Complementary therapies in pediatrics: a legal perspective Pediatrics 2005 115 3 774 780 10.1542/peds.2004-1093 15741385

[B21] Howlett M Ramesh M Studying Public Policy: Policy Cycles and Policy Subsystems 1995 1 Toronto, Ontario, Canada: Oxford University Press

[B22] Phidd R Canadian Public Policy: Ideas, Structures and Process 1992 2 Toronto, Ontario, Canada: Nelson Publishers

[B23] Grimaldi D Integration of complementary and alternative medicine into mainstream health care J Psychosoc Nurs Ment Health Serv 2008 46 10 8 9 18935930 10.3928/02793695-20081001-04

[B24] Meeker WC Public demand and the integration of complementary and alternative medicine in the US health care system J Manipulative Physiol Ther 2000 23 2 123 126 10.1016/S0161-4754(00)90081-2 10714541

[B25] Fink S International efforts spotlight traditional, complementary, and alternative medicine Am J Public Health 2002 92 11 1734 1739 10.2105/AJPH.92.11.1734 12406796 PMC3221476

[B26] World Health Organization National policy on traditional medicine and regulation of herbal medicine: Report of a WHO global survey 2005 WB 925 1 143

[B27] Gruenwald J Herzberg F The Global Nutraceuticals Market Business Briefing: Innovative Food Ingredients 2002 28 31

[B28] Ceylan S Hamzaoglu O Komurcu S Beyan C Yalcin A Survey of the use of complementary and alternative medicine among Turkish cancer patients Complement Ther Med 2002 10 2 94 99 10.1054/ctim.2002.0527 12481957

[B29] Gozum S Arikan D Buyukavci M Complementary and alternative medicine use in pediatric oncology patients in eastern Turkey Cancer Nurs 2007 30 1 38 44 10.1097/00002820-200701000-00007 17235218

[B30] AlBraik FA Rutter PM Brown D A cross-sectional survey of herbal remedy taking by United Arab Emirate (UAE) citizens in Abu Dhabi Pharmacoepidemiol Drug Saf 2008 17 7 725 732 10.1002/pds.1591 18395880

[B31] Al-Faris EA Al-Rowais N Mohamed AG Al-Rukban MO Al-Kurdi A Balla Al-Noor MA Al-Harby S Sheikh A Prevalence and pattern of alternative medicine use: the results of a household survey Ann Saudi Med 2008 28 1 4 10 10.4103/0256-4947.51761 18299652 PMC6074229

[B32] Klassen T LeBlanc S Methodological issues in research on public policy: Utilizing interviews Society 1993 17 2 21 26

[B33] Strauss A Corbin J Basics of Qualitative Research Techniques and Procedures for Developing Grounded Theory 1998 2 312

[B34] Ritchie J Spencer L Huberman M, Miles M Qualitative data analysis for applied policy research The Qualitative Researcher's Companion 2002 First Thousand Oaks: Sage Publications 305 32921884488

[B35] Ulin P Robinson E Tolley E Qualitative Methods in Public Health: A field guide for applied research 1993 San Francisco: Jossey-Bass

[B36] Kellehear A The Unobtrusive Researcher: A Guide to Methods 1993 Sydney, Australia: Allen and Unwin

[B37] Al-Mustaqbal newspaper Syndicate of physicians and order of pharmacists criticize the way herbal products are advertised Al-Mustaqbal newspaper 2007 Business(2559) 12 12

[B38] World Health Organization Regulatory Situation of Herbal Medicines: A worldwide review 1998 1 39 WHO/TRM/98.1

[B39] World Health Organization Guidelines on minimum requirements for the registration of herbal medicinal products in the Eastern Mediterranean Region 2006 1 27 WHO-EM/EDB/048/E/03.06/1000

